# Totally endoscopic atrial septal defect repair using transthoracic aortic cannulation in a 10.5-kg-boy

**DOI:** 10.1016/j.ijscr.2018.09.054

**Published:** 2018-10-10

**Authors:** Huy Q. Dang, Huong T. Le, Linh T.H. Ngo

**Affiliations:** a Minimally Invasive Cardiac Surgery Unit, Cardiovascular Center, Hanoi Heart Hospital, Hanoi, Viet Nam; b Department of Cardiovascular and Thoracic Surgery, Cardiovascular Center, E Hospital, Hanoi, Viet Nam

**Keywords:** Direct aortic cannulation, CO_2_ insufflation, Atrial septal defect, Minimally invasive cardiac surgery, Totally endoscopic surgery

## Abstract

**Introduction:**

Although totally endoscopic surgery (TES) has been widely applied for the treatment of atrial septal defect (ASD), small children receive few benefits from this technique due to risks of the femoral cannulation.

**Case presentation:**

A 23-month-old boy, weighing 10.5 kg, with the diagnosis of sinus venosus ASD underwent successful repair by TES. We performed this surgery through 4 small trocars (one 12 mm trocar and three 5 mm trocars), without robotic assistance. In this case, we inserted the arterial cannula directly into the ascending aorta instead of the femoral artery (FA). The defects were repaired on the beating heart with CO_2_ insufflation.

**Discussion:**

Femoral cannulation in small children pose some risks, such as increased arterial line pressure, critical lower limb ischaemia, and post-operative iliac or femoral arterial stenosis. Putting the arterial cannula directly into the ascending aorta is a good solution but is difficult to be performed through TES, especially in small children. The major concern of operating on the beating heart is the air embolism, which requires special preventative methods.

**Conclusion:**

Transthoracic aortic cannulation may facilitate TES in small children. However, the safety and efficacy of this approach needs to be validated by larger studies preferably randomised controlled trials.

## Highlights

• The scope of totally endoscopic cardiac surgery in children is limited.
• Femoral cannulation has risks which increase in small children.
• Aortic cannulation is the solution to avoid vessel complications in small children.
• With this technique, small children receive benefit from totally endoscopic surgery.

## 1 Introduction

Chest deformity is the major concern of sternotomy or thoracotomy in children [1]. In the treatment of ASD, the application of totally endoscopic surgery (TES) is limited by the weight of the patients. While some authors choose 20 kg to be the weight threshold [2], Wang et al. [3] and us [4] reported successful operation for children weighing from 13.5 kg. The smaller the patients are, the higher risks of femoral vascular complications they get. Theoretically direct aortic cannulation is a good solution for applying TES in small children. However, reports on this technique are limited. In this paper, we described a boy who weighed much lower than the threshold mentioned above, with the diagnosis of inferior type of sinus venosus ASD, was successfully operated on by TES, on the beating heart, with direct aortic cannulation. The work has been reported in accordance with the SCARE criteria [5].

## 2 Case report

An asymptomatic 23-month-old boy, weighed 10.5 kg, was incidentally diagnosed with congenital heart disease while presenting to the hospital for another illness. Transthoracic echocardiography (TTE) revealed one 18-mm ASD located in the inferior portion of the atrial septum that resulted in an overriding inferior vena cava (IVC), and the right inferior pulmonary vein (RIPV) partially returned to the right atrium (RA) near the orifice of the IVC. TTE also showed a complete left-to-right atrial shunt, no tricuspid regurgitation, and right ventricular dilation (with a diameter of 15 mm). Cardiac catheterization confirmed a normal anatomy of coronary arteries and a pulmonary to systemic flow ratio (Qp/Qs) of 3.2:1.

The patient was placed in a supine position with the right side of the body elevated to 30°. Two arms were placed along the body and the patient was under general anesthesia with a single-lumen endotracheal tube. One 14F-arterial cannula (Medtronic, Inc., Minneapolis, Minn, USA) used as a superior vena cava (SVC) cannula was inserted through the right internal jugular vein with Seldinger technique. Four trocars were set up on the right chest wall, included the following: one 12 mm trocar in the 5th intercostal space (ICS) at the anterior axillary line as the main working port, one 5 mm trocar in the 4th ICS at the mid-axillary line as the secondary working port, one 5 mm trocar in the 5th ICS at the mid-axillary line as the camera port and one 5 mm trocar in the 6th ICS at the mid-axillary line for right heart sucker.

The ventilation volume was reduced to 50%–75% compared with conventional practice. The anesthetist continuously monitored the oxygen saturation with a finger pulse oximeter and maintained it ≥95% throughout the operation. With this ventilation technique, the lungs were collapsed enough for the surgeon to open and hang up the pericardium. The large right lobe of the thymus covered the majority of the pericardium surrounding the aorta and the SVC. Therefore, we dissected this lobe from the pericardium (while preserving the tissue and supplying vessels) and hung it on to the anterior chest wall with a suture. The pericardium was opened parallel to and at 1.5 cm away from the anterior chest wall. The inferior edge of the pericardium was hung up to the diaphragm (the caudal end) and through the trocar (the cephalic end) by some sutures to expose the surgical field (Video 1). At this stage, respiratory ventilation was continued as usual.

**Fig. 1:**
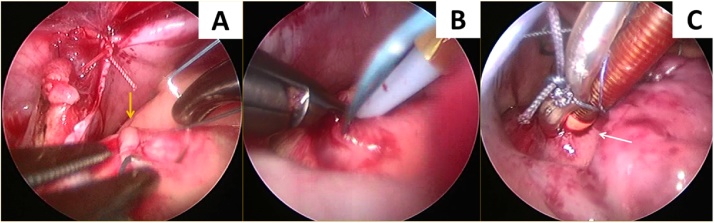
Aortic cannulation through the chest wall into the ascending aorta. **A**, a purse-string suture was made with a braided suture on the anterior wall of the ascending aorta, right beneath the semicircular fat plica (yellow arrow); **B**, a 12F-arterial cannula which was placed superiorly through right anterior chest wall in the 4^th^ ICS, was directed into the ascending aorta inside the purse-string suture; **C**, introduce the arterial cannula into the ascending aorta until the brake on the cannula reached the aortic wall. **ICS**, intercostal space.

**Fig. 2:**
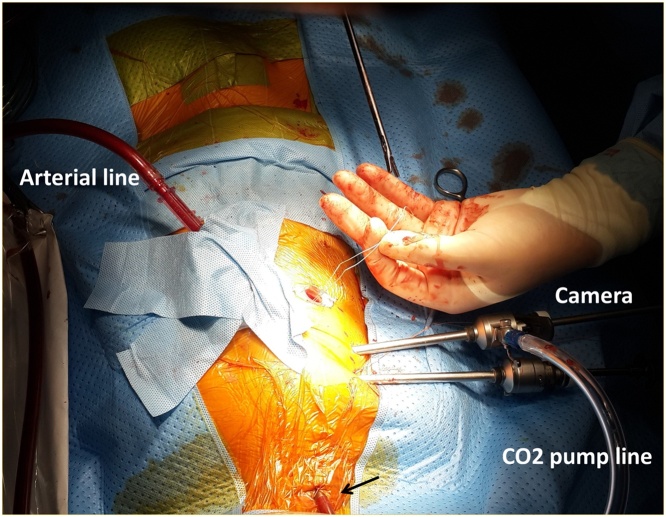
Cannulae and trocars set up on the chest wall. SVC cannula was inserted via the right jugular vein (black arrow), an arterial cannula was placed through the chest wall into the ascending aorta. One 12 mm trocar was used for main surgical field, three 5 mm trocars, CO_2_ pumping line was connected to the camera port. **SVC**, superior vena cava.

To expose the ascending aorta, the top of the right atrial appendage was sutured and pulled down through a trocar. A 2–0, 17 mm braided suture (ETHIBOND EXCEL^®^ Polyester Suture, ETHICON, JOHNSON & JOHNSON, Shanghai, China) was used to make a purse-string suture on the anterior wall of the ascending aorta, right beneath the semicircular fat plica (Fig. 1A) (Video 2). A 12F-arterial cannula (Medtronic, Inc., Minneapolis, Minn, USA) was placed superiorly through right anterior chest wall in the 4th ICS, 1 cm away from the right border of the sternum. This process was performed from the outside combined with endoscopic visualization from inside to avoid injury to the internal thoracic artery and ensure that the cannula was best directed to the purse-string suture (Fig. 2). We placed a piece of a 10 F rubber catheter (Red Rubber Latex All-Purpose Intermittent Catheters, Medline, USA) about 1.3 to 1.5 cm away from the tip of the arterial cannula to work as a brake. Subsequently, a surgical scalpel blade No.11 (Aesculap, Inc.) was used to open the ascending aorta inside the purse-string suture. The arterial cannula was then introduced via this ostium into the ascending aorta until the brake on the cannula reached the aortic wall (Fig. 1B, C) (Video 3). The arterial cannula was fixed and the cardiopulmonary bypass (CPB) was started.

A CO_2_-pump line connecting to the camera port (Fig. 2) was used to fill the pericardial and pleural spaces with CO_2_. Initially, CO_2_ was pumped with a rate of 0.5 l/min, and then the pump rate was adjusted to maintain the partial pressure of CO_2_ in arterial blood ranging from 35 to 40 mmHg. Arterial line pressure was maintained >50 mmHg during the operation.

**Fig. 3:**
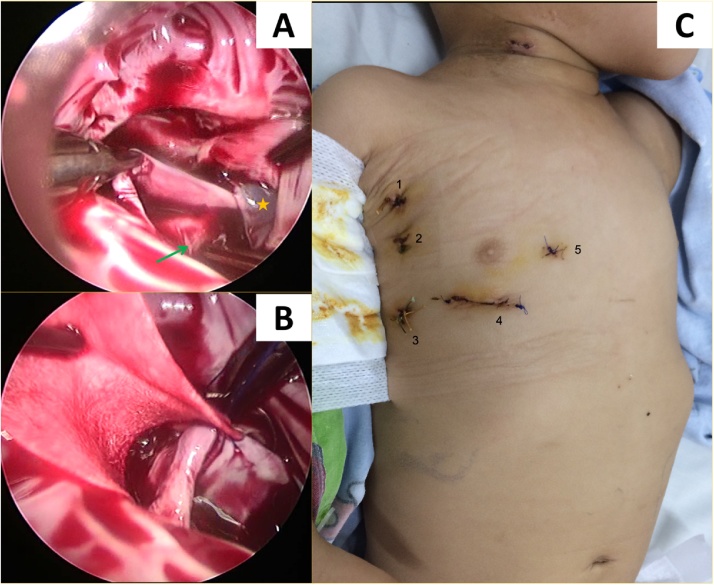
Intra- and postoperative images. **A**, Inferior type SV-ASD: the superior edge of the defect was retracted, the ASD located in the inferior portion of the atrial septum that led to an overriding IVC. The defect was partially covered by the Eustachian valve (yellow star) and the RIPV (green arrow) partially returned to the RA near the IVC orifice; **B**, close ASD with an artificial patch; **C**, surgical scars prior to discharge, include (1): secondary working port, (2): camera port, (3): port for right heart sucker, (4): main working port (12 mm trocar), and (5): hole for aortic cannulation. **SV-ASD**, sinus venosus atrial septal defect; **ASD**, atrial septal defect; **IVC**, inferior vena cava; **RIPV**, right inferior pulmonary vein; **RA**, right atrium.

A loop was placed around the SVC to act as a tourniquet but not snaring. The patient was placed in the Trendelenburg position. The tourniquet on the SVC was tightened after opening the RA (Video 4). The blood returning to the RA from the IVC was drained by a stiff sucker, which also acted as an atrial retractor to expose the lesion. The edges of RA were hung to the pericardium by stitches to expose structures inside the RA. After determining the location, size of the ASD, as well as the anatomical correlation between the IVC and the RIPV, an artificial patch was used to close the ASD and form a canal to drain blood from the RIPV to the LA through the ASD (Fig. 3A, B) (Video 5). Right before completing the ASD closure, the lung was inflated to remove air from the left atrium. The RA was closed in a two-layer fashion using continuous stitches. The extracorporeal circulation was stopped and the surgery was finished uneventfully. The operative and cardiopulmonary bypass times were 259 and 133 min, respectively. The patient stayed in the intensive care unit for 18 h and was discharged on postoperative-day 7 without neurological complication or blood transfusion. TTE prior to discharge revealed a completely closed ASD, patent IVC, and RIPV ostia. Both the patient and his family were extremely satisfied with the cosmetic results of surgical scars (Fig. 3C).

## 3 Discussion

Peripheral CPB is the standard technique in TES. There are two ways of inserting the femoral arterial cannulae: (1) directly and (2) indirectly through an artificial vessel. Most authors used direct cannulation [3,6]. This technique may shorten cannulation time while predisposing the patients to certain risks: (1) the pressure of the arterial line may increase gradually during the operation due to the reflex arterial spasm, especially in children, (2) acute lower limb ischeamia during and after surgery, and (3) postoperative stenosis of the iliac or femoral arteries. The smaller the patients are and the longer the operation time is, the risks are higher. In another report, we used a Knitted Dacron graft (Vascutek Terumo, Bangkok, Thailand) to connect to the common FA of the patient with an end-to-side anastomosis [4,7]. At the end of the operation, the graft was cut as near as possible to the anastomosis. The remains of the graft was closed simply. This method completely eliminated the risks of the leg ischaemia and the postoperative arterial stenosis. Regardless of the method of choice, the body weight cut-off point for TES in small children is 13.5 kg [3,4].

Another solution to ensure the flow and arterial pressure is aortic cannulation instead of femoral cannulation. Theoretically, directly inserted aortic cannula is the optimal method due to good arterial line pressure while avoiding vascular complications of the FA. In clinical practice, however, the direct aortic cannulation through small trocars is of extreme difficulty, especially in small children due to the following reasons: (1) the ventilation of the right lung must be decreased to help the surgeons to open the pericardium and expose the aorta while the anesthetist cannot use double-lumen endotracheal tube for these small patients, and (2) the surgeons have to insert the cannula into a small ascending aorta using endoscopic instruments without CPB support.

The IVC was not cannulated, blood from the lower part of the body (the flow was not too high due to small total body weight) was suctioned by a stiff sucker.

The major concern when performing surgery on the beating heart is air embolism [8]. We prevent this complication based on the following rules: (1) maintaining the arterial line pressure >50 mmHg throughout the repair of ASD, (2) filling the pericardial and the pleural space with CO_2_ with a pump rate of 0.5 l/min, and (3) inflating the lung to remove the air from the left atrium when completing the repair. The use of CO_2_ in surgery on the beating heart has been shown to play an important role in preventing air embolism and can replace aortic root needle [9,10]. These rules have been applied successfully in >120 patients diagnosed with ASD undergoing TES without aortic root needle.

## 4 Conclusion

Transthoracic aortic cannulation may facilitate TES in small children. However, the safety and efficacy of this approach needs to be validated by larger studies preferably randomised controlled trials.

## Conflicts of interest

No conflict of interest declared.

## Funding source

No funding was received for the study.

## Ethical approval

Ethical approval is not needed in Vietnam.

## Consent

Written informed consent was obtained from the parents of the patient for publication of this case report and accompanying images. A copy of the written consent is available for review by the Editor-in-Chief of this journal on request.

## Author contribution

All authors: dr. Dang, dr. Le and dr. Ngo have taken part in conception of the study, drafting and revising the whole manuscript critically. All authors have given their final approval of the manuscript upon submission.

## Registration of research studies

None.

## Guarantor

Huy Q. Dang.

## Provenance and peer review

Not commissioned, externally peer reviewed.
